# mTOR Promotes Tissue Factor Expression and Activity in EGFR-Mutant Cancer

**DOI:** 10.3389/fonc.2020.01615

**Published:** 2020-08-14

**Authors:** Ying Cong, Qingrou Li, Xuesai Zhang, Yaqing Chen, Ker Yu

**Affiliations:** Department of Pharmacology, School of Pharmacy, Fudan University, Shanghai, China

**Keywords:** mTOR inhibitor, tissue factor, tumor microenvironment, EGFR mutant cancer, mTOR-TF axis

## Abstract

Mechanistic target of rapamycin (mTOR) signaling pathway mediates the function of oncogenic receptor tyrosine kinases (RTKs). We aimed to elucidate new role of mTOR in EGFR-mutant (EGFR-mut) non-small cell lung cancer (NSCLC) and glioblastoma (GBM) with a focus on tumor microenvironments. Here, we report a novel regulatory link between mTOR complexes (mTORCs) and tissue factor (TF), an initiator of tumor-derived thrombosis. TF is elevated in EGFR-mut NSCLC/GBM cell lines and tumors from patients with poor prognosis. Application of mTORC1/2 inhibitors (AZD8055, WYE-125132, MTI-31, and rapamycin) or genetic mTORC-depletion all reduced TF expression, which appeared to be differentially mediated depending on cellular context. In U87MG and HCC827 cells, mTORC1 exerted a dominant role via promoting TF mRNA transcription. In EGFR-TKI-resistant H1975 and PC9 cells, it was mTORC2 that played a major role in specific repression of lysosomal-targeted TF protein degradation. Successful inhibition of TF expression was demonstrated in AZD8055- or MTI-31-treated H1975 and U87MG tumors in mice, while a TF-targeted antibody antagonized TF activity without reducing TF protein. Both the mTOR- and TF-targeted therapy induced a multifaceted remodeling of tumor microenvironment reflecting not only a diminished hypercoagulopathy state (fibrin level) but also a reduced stromal fibrosis (collagen distribution), compromised vessel density and/or maturity (CD31 and/or α-SMA) as well as a substantially decreased infiltration of immune-suppressive M2-type tumor-associated macrophages (CD206/F4/80 ratio). Thus, our results have identified TF as a functional biomarker of mTOR. Downregulation of mTOR-TF axis activity likely contributes to the therapeutic mechanism of mTORC1/2- and TF-targeted agents in EGFR-mut advanced NSCLC and GBM.

## Introduction

The signaling pathway of the mechanistic target of rapamycin (mTOR) is one of the most important signaling network downstream of the oncogenic receptor tyrosine kinases (RTKs), which participate in tumor growth, metastasis and therapy evasion (1, 2). mTOR exists as two molecularly distinct protein complexes termed mTORC1 and mTORC2, which differentially and coordinately function in a multitude of biological processes (1, 2). In non-small cell lung cancer (NSCLC) or glioblastoma where EGFR driver mutation (EGFR-mut) and/or gene amplification are prevalent, mTOR activation contributes to disease progression, metastasis and tyrosine kinase inhibitor (TKI) resistance (3–6). Recent studies also suggest that mTOR not only promotes intrinsic tumor cell growth and survival property, but may also contributes to regulation of an unfavorable tumor microenvironment that may further hinder treatment outcome (7–9). However, the molecular underpinnings and/or functional mediators of mTOR complexes in disease progression, and especially in tumor microenvironment conformation, remains poorly understood. We recently demonstrated that in EGFR-mut and EGFR-TKI resistant NSCLC preclinical models, mTORC2 is critically required for epithelial-mesenchymal transition (EMT) and tumor growth in the mouse brain in a brain-borne microglia/macrophage-dependent manner (10). To expand our knowledge in this area, we are interested in searching for new and previously uncharacterized component(s) of the mTOR signaling network in advanced EGFR-mut cancers.

Tissue factor (TF) is a 47 kDa transmembrane glycoprotein that normally acts as an initiator of body’s extrinsic coagulation process. TF binds to Factor VII to trigger a cascade of proteases leading to thrombin activation and fibrin clot formation (11). Aberrant expression of TF, however, is reported in many aggressive tumors and is the basis for tumor-initiated thrombosis, increased metastatic properties and poor disease prognosis (12–17). TF may directly or indirectly contribute to tumor progression through actions on tumor microenvironment (18–22). Nevertheless, it remains unclear how TF expression and function are regulated in EGFR-mut cancers, particularly in the EGFR-TKI-resistant NSCLC tumors. It is also unclear about the potential TF relationship with commonly known oncogenic pathways. It was previously reported that TF expression can be stimulated by EGF in glioma cells (23). Given the critical involvement of mTOR in EGFR-mut cancers, it is possible that TF might be a molecular and functional target of mTOR complexes in these tumors.

In this study, we employ various genetic and pharmacology approaches to elucidate that TF is elevated in EGFR-mut lung and brain cancers in an mTOR-dependent manner. mTORC1 and mTORC2 act differentially and coordinately to promote TF expression mediated through distinct transcription and posttranslational mechanisms. The mTOR-TF axis functionally contributes to an unfavorable tumor microenvironment relevant for disease progression and therapy resistance. Targeting of the mTOR-TF axis may offer a new treatment strategy.

## Materials and Methods

### Chemicals and shRNA

Specific mTOR inhibitor AZD8055, WYE-125132 (WYE-132), Rapamycin and EGFR inhibitor AZD9291 were purchased from BioChemPartner (Shanghai). mTOR inhibitor MTI-31 was synthesized as described (24). Inhibitors were dissolved in DMSO as 20 mM stock solution and were diluted before assays. The anti-TF antibody SC1 was generated as described (25). The pGIPZ-based lentiviral shRNA for human Raptor (V3LHS_329849) or Rictor (V2THS_225915) and non-targeting (NT, RHS4346) were obtained from Open Biosystems/GE Dharmacon. Recombinant human EGF (PeproTech) and PS341 (MedChemExpress) were purchased. All other chemicals or general buffer reagents were purchased from Sigma-Aldrich unless specified.

### Cell Culture and Gene Knockdown

Cell lines of NCI-H1975 and U87MG were obtained from American Type Culture Collection (ATCC). HCC827 was obtained from the Cell Bank of Chinese Academy of Sciences (CAS). PC9 was obtained from European Collection of Cell Cultures (ECACC). Cells were cultured using standard cell culture methods and reagents (Invitrogen). All the cells were periodically tested for mycoplasma contamination free. Various pGIPZ-shRNA viruses were packaged in HEK293T before infecting tumor cells.

### Cell Lysates and Immunoblotting

Cells were seeded at appropriate density 1 day before drug treatment. Cells stimulated by EGF were starved with serum-free culture medium overnight. Various inhibitors (taken from 20 mM stock in DMSO) were first prepared as 100x concentrated solutions before being added to cell culture. Cells were treated for 24 h or as indicated, lysed in NuPAGE-LDS lysis buffer (Invitrogen) and immunoblotted with primary antibodies against TF (R&D, Cat#MAB2339), P-EGFR (Epitomics, Cat#1138-1), Raptor (Proteintech, Cat#20984-1-AP), Rictor (CST, Cat#2114), P-S6 (Epitomics, Cat#2268-1), P-AKT (Abcam, Cat#Ab81283), pERK (CST, Cat#4370), and GAPDH (Bioworld, Cat#AP0063).

### Real-Time qPCR

Total RNA was extracted by TRIZOL (Invitrogen) reagent following standard protocol. Reverse transcription and cDNA were performed using PrimeScript^TM^ RT Master Mix (Takara, No. RR036A). Real-time Quantitative PCR was performed by using SYBR Premix Ex Taq^TM^ (Takara, No. RR420A). The primer pairs for human TF were F: 5′-GGCGCTTCAGGCACTACAA-3′ and R: 5′-TTGATTGACGGGTTTGGGTTC-3′; for human GAPDH were F: 5′-ACAACTTTGGTATCGTGGAAGG-3′ and R: 5′-GCCATCACGCCACAGTTTC-3′.

### *In vivo* Tumor Growth Inhibition

*In vivo* efficacy studies were performed under protocols approved by institutional IACUC of Fudan University. Xenograft tumor models were established in female Balb/c nude mice (4–6 weeks old) by subcutaneous implantation with H1975 or U87MG (5 × 10^6^), respectively. Tumors were staged at an initial tumor volume of 150–200 mm^3^ and randomized into treatment groups (*n* = 8). Free-base form MTI-31 or AZD8055 were formulated in DMSO/2-Hydroxypropyl)-β-cyclodextrin (final vol/vol: 5%/19%) as clear solution. These agents were prepared twice weekly and dosed orally once daily (qd). mIgG and SC1 were formulated in PBS and dosed intravenously (i.v.) once weekly. Tumor volume was monitored using a caliper twice a week and calculated using the formula V = LW2/2 (where V, volume; L, length; and W, width).

### Tumor Immunohistochemistry (IHC) and Immunofluorescence (IF)

Tumors were snap-frozen in liquid nitrogen or formalin fixed for 24 h and embedded in paraffin. Tumor slides were deparaffinized, rehydrated, and permeabilized with 1% Triton X-100. Antigens were retrieved with Tris/EDTA (pH 9.0) or citrate antigen retrieval solution (pH 6.0) under microwave heating for 20 min, blocked with 5% goat serum in TBST. Slides were incubated with antibodies CD31 (Bioapsi Bioscience, Cat#BA9001), α-SMA (Servicebio, Cat#GB13044), F4/80 (Bioapsi Bioscience, Cat#BA9408), CD206 (Abcam, Cat#ab64693), fibrin (Abcam, Cat#ab34269), TF (SC1 generated in house), or Masson’s trichrome (Sigma, Cat#HT15-1KT). Images were acquired using Leica microscope (model DMI4000D) with 200X magnification. Representative views (1/8 size captured from the original photo) are shown. Eight view fields per tumor were assessed for quantification analysis. Positive areas were quantified by ImageJ and analyzed in GraphPad Prism software.

### Statistical Analysis

All numerical data are presented as mean ± standard deviation (SD) except for mouse xenograft study data which is presented as mean ± standard error (SE). Numerical data processing and statistical analysis were performed with Microsoft Excel and GraphPad Prism 6.01 software. *P*-values were calculated using the unpaired two-tailed Student *t*-test.

## Results

### TF Is Frequently Elevated in EGFR-Mutant Lung or Brain Cancer Lines and Tumor of Patients With Poor Prognosis

**FIGURE 1:**
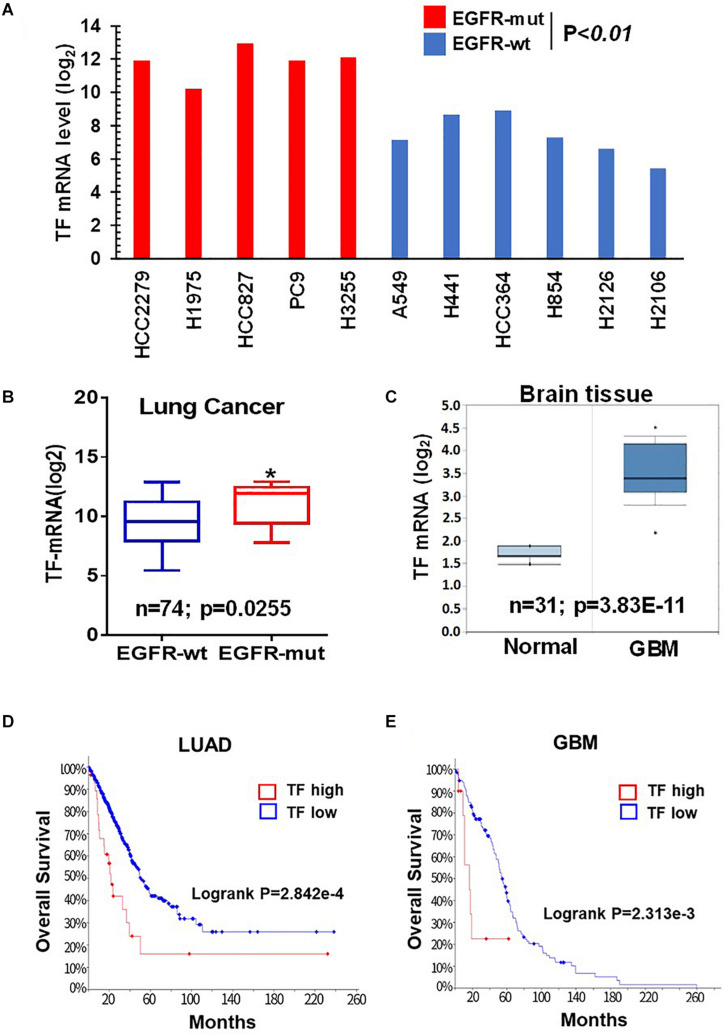
Tissue factor (TF) is elevated in EGFR-mutant NSCLC and GBM. **(A)** TF mRNA levels for EGFR-mut versus EGFR-wt NSCLC cell lines were analyzed from CCLE database. **(B)** TF mRNA levels for EGFR-mut versus EGFR-wt lung adenocarcinoma tumors were analyzed from TCGA (https://portal.gdc.cancer.gov; dataset accessed in September 2018). **(C)** TF mRNA levels for normal brain and GBM tissue were analyzed from Oncomine (https://www.oncomine.org; dataset accessed in 2017). **(D,E)** Overall survival rate (OS) was analyzed by using Kaplan-Meier on cBioportal (https://www.cbioportal.org; TCGA PanCancer Atlas dataset accessed in October 2019) for lung adenocarcinoma **(D)** and GBM **(E)**. *P < 0.05.

Given the critical role of mTOR in oncogenic EGFR signaling function, we considered the possibility that mTOR may promote TF expression in EGFR-mutant cancers. To begin testing this scenario, we first analyzed the Cancer Cell Line Encyclopedia (CCLE) database and found that TF mRNA level tends to be higher in the EGFR-mutant (EGFR-mut) lung cancer cell lines compared to that of EGFR-wild type (EGFR-wt) cell lines (Figure 1A). We then examined lung cancer tumor data from The Cancer Genome Alta (TCGA), which also revealed an elevated TF level in EGFR-mut tumors (Figure 1B). As human gliomas represent another mTOR pathway-activated cancer type arising from its frequent occurrence of EGFRvIII and/or loss of tumor suppressor PTEN (26), we also examined TF expression in these tumors. Analysis of Oncomine dataset demonstrated TF mRNA to be significantly higher in glioblastoma (GBM) compared to that of normal brain (Figure 1C). Furthermore, Kaplan-Meier analysis of patient overall survival (OS) data showed that patients with TF-high expressing lung tumors (Figure 1D) or GBM (Figure 1E) generally had a worse prognosis compared to that for TF-low patients. These results suggest that the aberrant TF expression in these tumors may contribute to disease progression and poor treatment outcome.

### mTOR Promotes TF Overexpression in EGFR-mut Tumors Through Coordinated Yet Differential Involvement of mTORC1 and mTORC2

**FIGURE 2:**
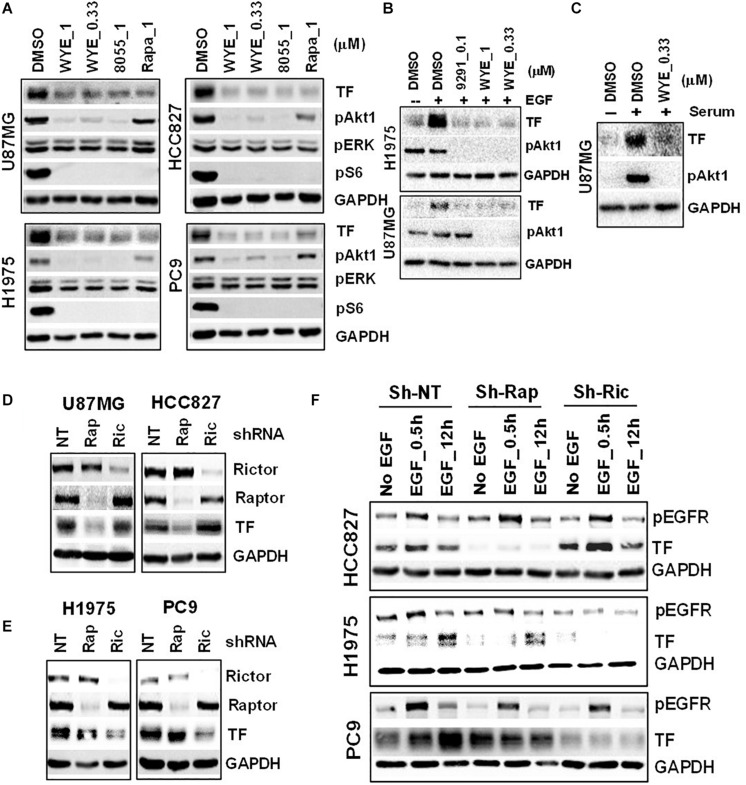
Mechanistic target of rapamycin (mTOR) promotes TF overexpression in EGFR-mut tumors. **(A)** mTOR inhibitor WYE-132 and AZD8055 decreased TF expression in four indicated cell lines. The indicated cells were treated for 24 h in full growth medium followed by immunoblotting. **(B)** The indicated cells were serum-starved then stimulated with 50 ng/mL EGF without or with inhibitor for 24 h. **(C)** Serum-starved U87MG cells were stimulated with 10% serum without or with WYE-132 for 12 h and immunoblotted. **(D,E)** The indicated cell lines were infected with pGIPZ-shRNA encoding lentivirus, incubated for 6–7 days in full-growth medium and immunoblotted. **(F)** Cells as in D and E were serum-starved, stimulated with EGF and immunoblotted.

To probe the regulation of TF by mTOR in EGFR-mut tumors, we chose three EGFR-mut lung cancer lines (HCC827, H1975, and PC9) and one GBM line harboring EGFRvIII and PTEN-loss (U87MG) as disease models for treatment with structurally distinct mTORC1/2 inhibitors (mTOR inhibitors) including Rapamycin, AZD8055, WYE-125132 (WYE-132), and MTI-31 (24, 27, 28). When these four cancer lines were cultured in full-growth media, treatment with these four independent mTOR inhibitors all resulted in a decrease in the steady state level of TF protein (Figure 2A; see below). In serum-starved cells, addition of EGF (Figure 2B) or serum (Figure 2C) each stimulated TF expression, which were largely prevented by co-treatment with mTOR inhibitors. We next performed gene depletion analysis to assess the role of mTOR complexes (mTORCs). In U87MG and HCC827 cells, depletion of Raptor (disrupting mTORC1) significantly reduced TF protein level while depletion of Rictor (disrupting mTORC2) had a minor effect (Figure 2D). In H1975 and PC9 cells, however, TF protein level reduced only in the mTORC2-disrupted cells (Figure 2E). To confirm these contrasting results, the cells were serum-starved and induced with EGF. Consistently, the reduction in basal- and EGF-stimulated TF levels were differentially observed in mTORC1-disrupted HCC827 cells but in mTORC2-disrupted H1975 or PC9 cells (Figure 2F). These results overall indicate that mTOR promotes TF expression in EGFR-mut/mTOR-activated lung cancer and GBM cells, which are mediated differentially through mTORC1 and/or mTORC2 in a cellular context-dependent manner.

### mTOR Inhibitor Differentially Targets TF-mRNA Transcription and/or TF Protein Degradation in a Cellular Context-Dependent Manner

**FIGURE 3:**
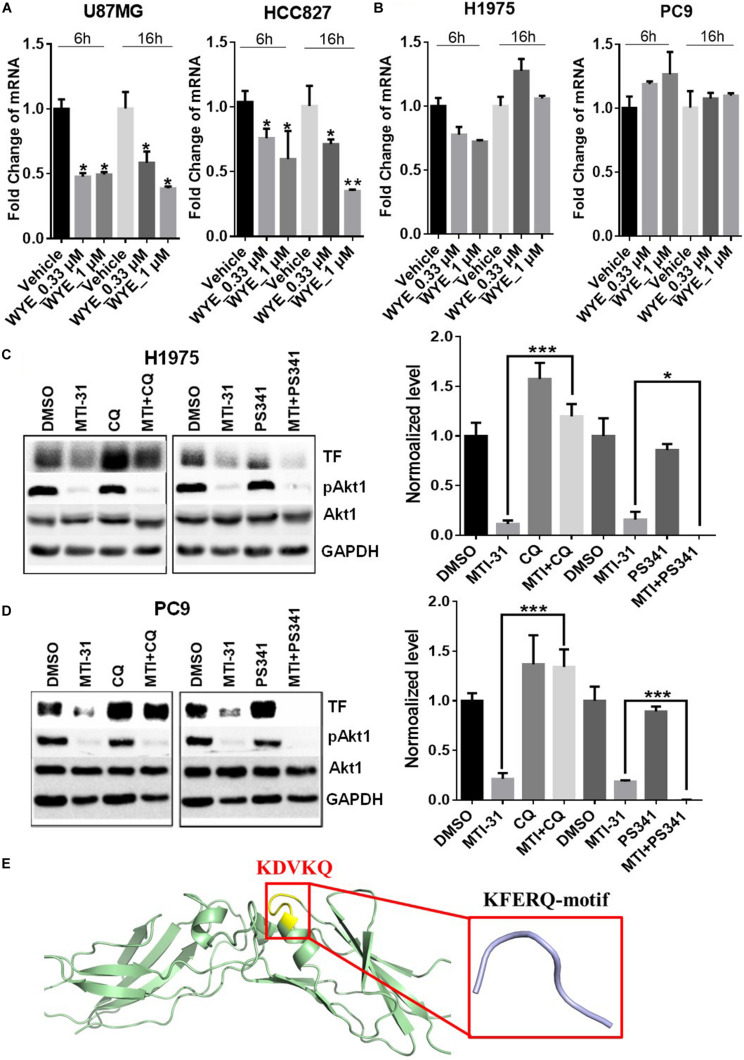
Mechanistic target of rapamycin (mTOR) inhibitor differentially targets TF-mRNA transcription or TF protein degradation in a cellular context-dependent manner. **(A,B)** U87MG and HCC827 **(A)**, H1975 and PC9 **(B)** cells were treated as indicated, subjected to qPCR analysis of TF mRNA as described in section “Materials and Methods.” The relative mRNA levels are shown. **(C,D)** H1975 **(C)** and PC9 **(D)** cells were treated with 5 μmol/L MTI-31 alone or in combination with 10 μmol/L CQ or 0.01 μmol/L PS-341 for 24 h, then immunoblotted (left panels) and quantified (right panels). *P < 0.05; **P < 0.01, ***P < 0.001. **(E)** The amino acid sequence of TF was download from https://www.ncbi.nlm.nih.gov/to set up the model of TF structure using Pymol software. The KFERQ-motif is marked in yellow and its sequence is marked in red.

To elucidate the mechanism of TF down-regulation in response to mTOR inhibitor, we performed quantitative polymerase chain reaction (qPCR) to examine if mTOR regulates TF mRNA transcription. Treatment of U87MG and HCC827 cells with mTOR inhibitor in full-growth media for 6 or 16 h resulted in a rapid and sustained decrease in TF mRNA levels (Figure 3A). However, similar treatment of H1975 and PC9 cells had minimal and variable effect on TF mRNA (Figure 3B). As U87MG/HCC827 and H1975/PC9 differ in relative contribution from mTORC1 versus mTORC2 in promoting TF expression (Figures 2D–F), we considered the possibility that mTORC2 may mediate TF protein stability in the mTORC2-dominant tumor cells like H1975 and PC9. To test this scenario, cells were treated with mTOR inhibitor MTI-31 alone, in combination with a proteasome inhibitor PS-341, or in combination with a lysosome acidification inhibitor chloroquine (CQ). Immunoblotting analysis showed that in both H1975 and PC9 cells, co-treatment with CQ near completely blocked the inhibitory effect of MTI-31 on TF, while co-treatment with PS341 failed to rescue from MTI-31 inhibition and may have possibly enhanced it (Figures 3C,D). Because the classical lysosome-mediated macroautophagy is regulated by mTORC1, we searched for a new mTORC2-dependent mechanism that may account for lysosomal degradation of TF. We found that mTORC2 is involved in suppression of the chaperone mediated autophagy (CMA) (29), which specifically target substrate proteins containing the KFERQ-like loop motif (30, 31). We therefore employed Pymol software to construct TF protein homology model. Sequence blast revealed the KFERQ-like motif KDVKQ located at amino acid 97–101 of TF protein expected to be recognized by chaperon (Figure 3E). We conclude from these results that mTOR inhibitors can target TF mRNA transcription and/or trigger TF protein lysosomal degradation in a cellular, context-dependent manner likely involving differential actions of mTORC1-sensitive TF gene transcription and/or mTORC2-sensitive CMA.

### Downregulation of TF Expression and Activity in EGFR-mut Tumors *in vivo*

**FIGURE 4:**
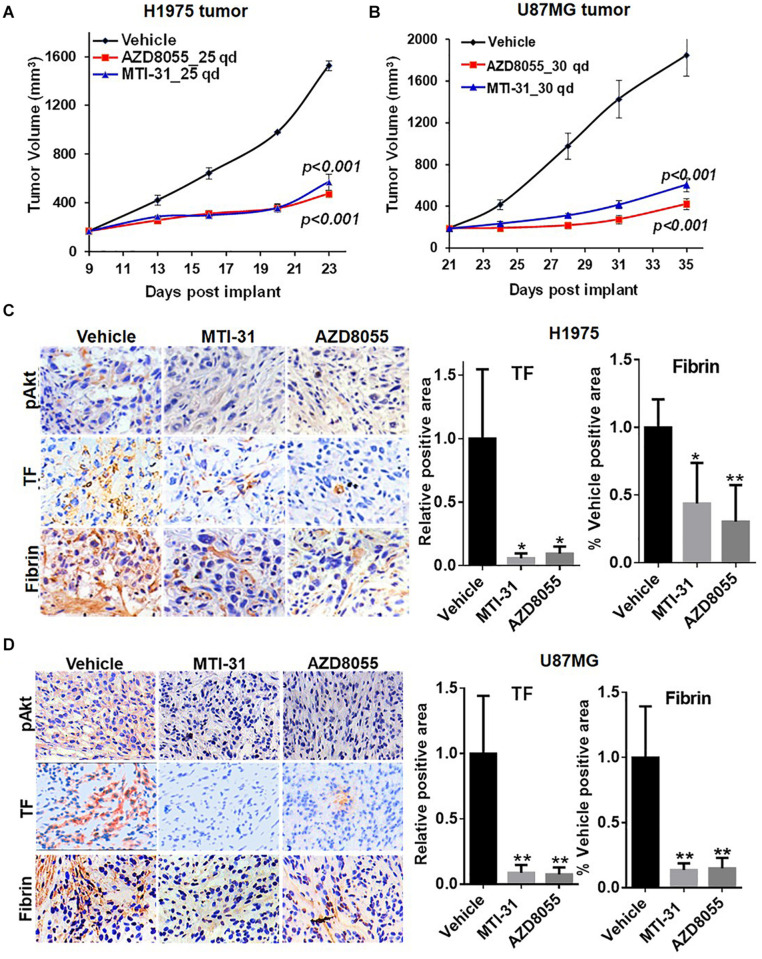
Downregulation of TF expression and activity in EGFR-mut tumors *in vivo*. **(A,B)** Studies of H1975 and U87MG tumor growth in nude mice and mTOR inhibitor treatment with MTI-31 or AZD8055 were performed as described in section “Materials and Methods.” **(C,D)** On final day of tumor growth studies, H1975 tumors **(C)** and U87MG tumors **(D)** were dissected and analyzed by IHC and/or IF as described in section “Materials and Methods.” Tumor slides were probed with anti-pAkt, anti-TF and anti-fibrin (left panels) quantified (right panels). *P < 0.05, **P < 0.01.

We next employed H1975 and U87MG *in vivo* tumor models to study mTOR inhibitor effects on TF expression and function. As expected, treatment of tumor bearing nude mice with orally administered MTI-31 or AZD8055 inhibited growth of H1975 tumors (Figure 4A) or U87MG tumors (Figure 4B). We performed staining of the tumor sections through immunohistochemistry (IHC) and/or immunofluorescence (IF) to assess mTOR-targeted inhibition of TF (Figures 4C,D). Suppression of mTOR activity was demonstrated by the decrease of mTOR downstream marker pAkt1 (S473) in treated H1975 and U87MG tumors (Figures 4C,D, left top panels). Importantly, mTOR inhibitors also reduced staining level of tumor-associated TF protein, which was consistent with our *in vitro* results (Figures 2A, 4C,D, left middle panels). Because TF initiates thrombin activation that in turn generates fibrin deposits (11), we then performed fibrin staining. Indeed, tumor-associated fibrin level was substantially decreased in treated H1975 and U87MG tumors (Figures 4C,D, left bottom panels). Taken together, these results demonstrate that mTOR regulates TF expression and TF activity in EGFR-mut tumors *in vivo*.

### Involvement of mTOR-TF Axis in Unfavorable Tumor Microenvironment

**FIGURE 5:**
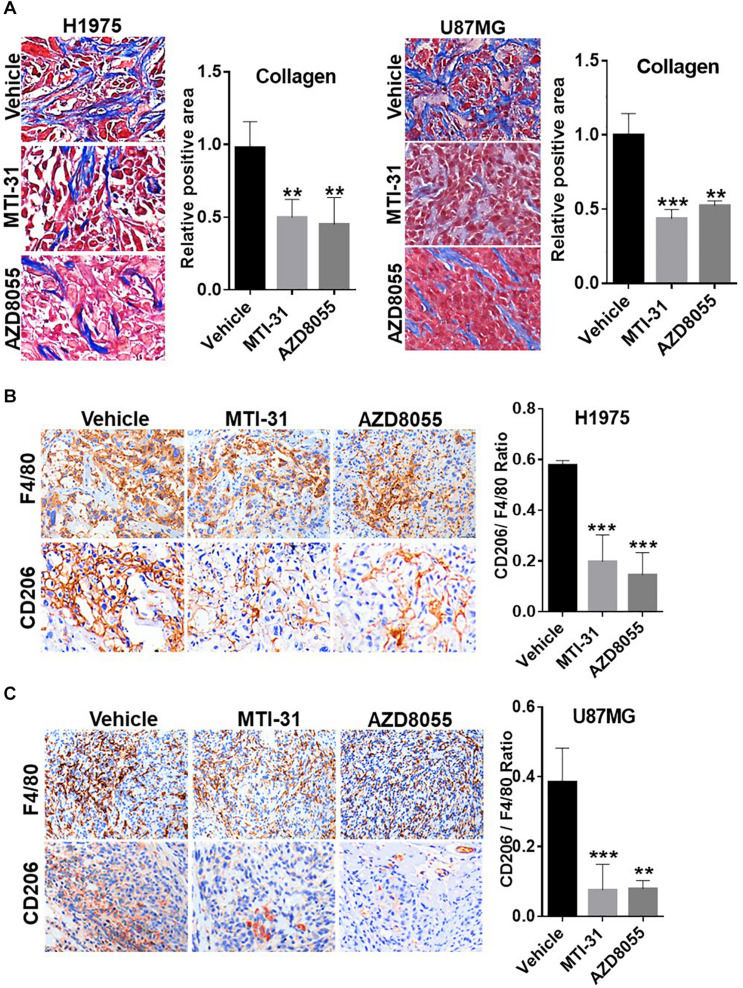
Involvement of mTOR-TF axis in tumor microenvironment. **(A)** H1975 and U87MG tumor sections as described in Figure 4 were subjected to Masson’s trichrome staining as described in section “Materials and Methods.” **(B,C)** Tumor sections as in **(A)** were analyzed for TAM infiltration by IHC staining with anti-F4/80 and anti-CD206 as described in section “Materials and Methods.” The calculated the ratio of CD206 versus F4/80 indicates the levels of M2-type TAMs. For **(A–C)**, representative images are shown (left panels) and quantified (right panels). **P < 0.01, ***P < 0.001.

Tumor microenvironment is increasingly relevant for disease progression and therapy response. We examined several established tumor-promoting components such as stromal collagen, tumor vasculature, and macrophage distribution (Figure 5). Collagen distribution was significantly reduced after mTOR inhibitor treatment (Figure 5A). IHC or IF staining of CD31 showed both reduced vessel density and vessel lumen size (Supplementary Figures S1A,B). As vessel integrity requires pericyte coverage of vascular sprouts, we also examined pericyte marker α-SMA. There was a significantly reduced pericyte coverage on CD31^+^ tumor vessels of mTOR inhibitor-treated tumors (Supplementary Figures S1A,B). Tumor associated macrophages (TAMs), particularly the M2-type TAMs, are multifunctional immunosuppressive cells linked to tumor progression (32). We analyzed TAMs profile by staining F4/80 (macrophage pan-marker) and CD206 (M2-type). While mTOR inhibitor did not reduce the total F4/80 level, the ratio of CD206 versus F4/80 was substantially reduced indicating a specific inhibition of M2-type TAMs (Figures 5B,C). The results in Figure 5 suggest that the mTOR-targeted TF inhibition may improve tumor microenvironment compatible with higher stromal permeability, less angiogenic, and more susceptible to therapy treatment.

### TF-Targeted Antibody Induces Tumor Microenvironment Remodeling

**FIGURE 6:**
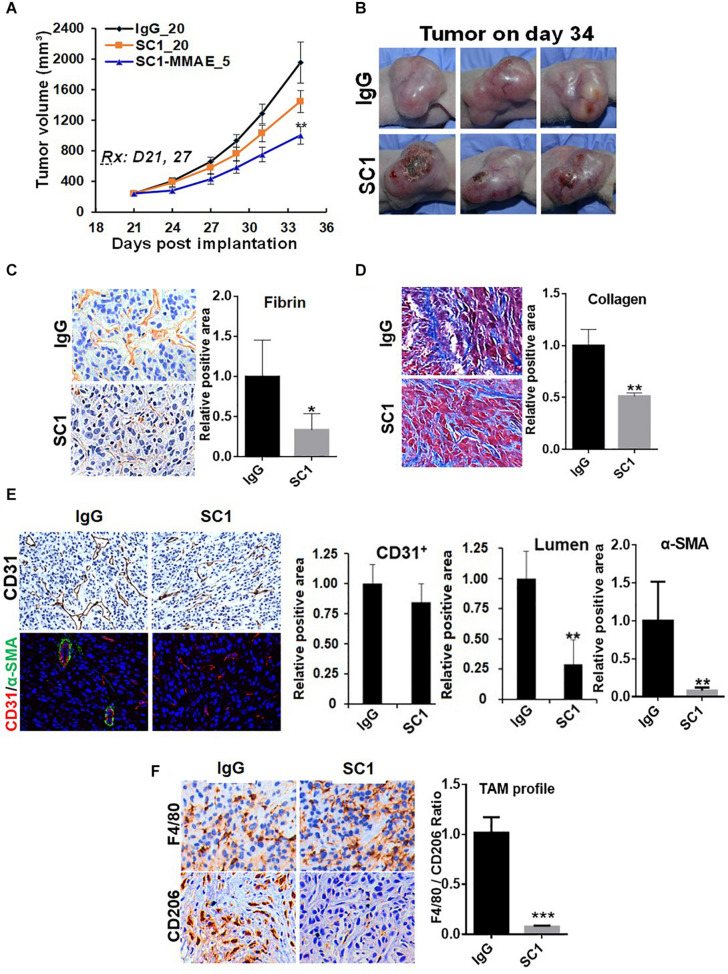
TF-targeted antibody induces tumor microenvironment remodeling. **(A)** Nude mice bearing established U87MG tumors (*n* = 8) were treated with 20 mg/kg IgG or SC1, 5 mg/kg SC1-MMAE i.v. 1x weekly as indicated for a total of two treatments. Tumor growth curves are plotted. **(B)** Significant necrosis was observed in SC1-treated tumors. Tumor images are shown. **(C,D)** Fibrin deposits served as TF activity biomarker **(C)** and collagen distribution **(D)** were stained by IHC as described in section “Materials and Methods.” **(E)** Tumor blood vessels were analyzed by IHC with anti-CD31 or IF co-staining with anti-CD31 and anti-α-SMA. CD31-positive/lumen area and α-SMA-positive area in CD31^+^ vessels were quantified. **(F)** TAM infiltration was tested by IHC staining with anti-F4/80 and anti-CD206 similarly as Figure 5. The calculated the ratio of CD206 versus F4/80 indicates the levels of M2-type TAMs. For **(C–F)**, representative images are shown (left panels) and quantified (right panels). *P < 0.05, **P < 0.01, ***P < 0.001.

To further study the functional relationship of mTOR and TF in these tumors, we investigated the effects of an anti-TF antibody SC1 on tumor microenvironment. Previously, depletion of TF gene or treatment with SC1 in TF-high expressing pancreatic or breast cancer cells did not acutely block cell proliferation *in vitro*, but elicited *in vivo* efficacy in nude mice xenograft tumor model (25). In this study, co-implantation of SC1 with U87MG cells (Supplementary Figure S2) or intravenously treated U87MG tumor-bearing mice with suboptimal dose regimen of SC1 (Figure 6A) showed a clear trend for efficacy, although the tumor volume reduction did not reach statistical significance. Meanwhile, we found that tumor necrosis occurred much more extensively in SC1-treated tumors compared to that of IgG-control tumors (Figure 6B). IHC and IF analyses of tumor sections revealed a reduction in the TF activity marker fibrin (Figure 6C) and a decreased stromal collagen distribution (Figure 6D), which are consistent with mTOR inhibitor-treated tumors (Figures 4C,D, 5A). In SC1-treated tumors, although the overall vascular density (CD31^+^ staining) was not significantly decreased, there existed many collapsed vessels correlating a diminished level of α-SMA staining on CD31^+^ vessel structures (Figure 6E). These results are largely in line with those of mTOR inhibitor treatment, but differ in that SC1 mainly affected vessel maturation without direct inhibition of CD31-dependent endothelial proliferation. Like the mTOR inhibitor, SC1-treated tumors displayed a substantially reduced level of CD206 without affecting F4/80, indicating a strong suppression of M2-type TAMs (Figure 6F). Collectively, the results in Figures 4–6 support the notion that TF is a functional target of mTOR pathway in these tumors. Pharmacological targeting of the mTOR-TF axis improves tumor microenvironment for therapy response.

## Discussion

While tumor-initiated thrombosis has been long recognized for cancer related patient mortality (33), the role of hypercoagulable state in tumorigenesis and development represent an emerging area of cancer research (12–17). As a key initiator of tumor-derived hypercoagulopathy, TF is aberrantly expressed in many types of aggressive tumors and may be highly relevant for understanding the disease mechanism and treatment outcome. The importance of TF is also supported by more recent findings with thrombin, a critical TF-cascade protease that impacts tumor progression and cancer immune evasion (34, 35).

Our current study uncovered a novel regulatory network between mTOR and TF in cancer development. We revealed that TF is elevated in EGFR-mut NSCLC or GBM and correlates with worse patient survival. In our panel of cancer cells with EGFRvIII (U87MG), EGFR-mut NSCLCs exhibiting gefitinib (TKI)-sensitive (HCC827), partially resistant (PC9), and resistant (H1975), treatment with several specific and distinct mTOR inhibitors all led to significant inhibition in both basal- and EGF-stimulated level of TF expression. The mechanism of TF-inhibition appeared to be differentially regulated depending on cellular context. In U87MG and HCC827 cells, mTORC1 exerted a dominant role in promoting TF expression, which primarily involved TF mRNA transcription.

In the TKI-resistant H1975 and PC9 cells, it was mTORC2 that played a major role in promoting the elevated TF expression, while depletion of mTORC1 had no effect. Treatment of these cells with mTOR inhibitor did not significantly reduce TF mRNA transcription; rather, the TF inhibition was mediated through a specific lysosomal-targeted degradation pathway. Interestingly, it was recently identified that a particular form of lysosomal-targeted protein degradation, termed CMA (chaperone-mediated autophagy), is specifically inhibited by mTORC2/AKT (29), which is mechanistically different from the mTORC1-regulated classical macroautophagy pathway. In this regard, we previously made similar observations for mTORC2-targeted loss of hexokinase 2 (HK2) in HER2-mut breast cancer cells (not shown). HK2 is a validated CMA substrate (36). Employing bioinformatics tools, we revealed the presence of a conserved KFERQ-like CMA-targeting motif within TF protein. While it requires further validation of TF as a bona fide substrate of CMA, it is interesting to speculate that activation of mTORC2/AKT axis in subsets of EGFR-mut and TKI-resistant NSCLC cells may perturb the physiological balance of CMA activity leading to aberrant accumulation of malignancy-promoting targets such as TF.

Tissue factor is a functional target of mTOR in tumor microenvironment. This conclusion is based on several common improvements observed with tumors treated with mTOR inhibitors or the TF-targeted antibody. This overlapping action profile is consistent with TF as a downstream mediator of mTOR. Successful inhibition of TF expression was demonstrated in MTI-31/AZD8055-treated H1975 and U87MG tumors while TF antibody, as expected, antagonized TF signaling activity without reducing TF protein level (25). Both the mTOR- and TF-targeted therapy induced a multifaceted remodeling of tumor microenvironment, reflecting not only a diminished hypercoagulable state (fibrin level), but also a reduced stromal fibrosis (collagen distribution), compromised vessel density and/or maturity (CD31 and/or α-SMA) as well as a substantially decreased infiltration of M2-type TAMs (CD206/F4/80). The decreases in stromal fibrosis and tumor blood vessel function will significantly aid antitumor efficacy. While mTOR is known for regulating angiogenesis, the mTOR-TF axis appears to selectively modulate vessel maturity via pericyte function. The M2-type TAMs are tumor-promoting, immune-suppressive, regulatory cells and are frequently found in NSCLC tumors (32). The inhibition of M2-type TAMs in EGFR-mut tumors is significant and may enhance efficacy of existing cancer immunotherapy.

Lastly, TF inhibition leads to reduced thrombin and fibrin generation. Like TF, thrombin triggers clotting and also promotes cellular events through protease-activated receptors (PARs) leading to cancer metastasis (37, 38). In our experience, disruption of mTORC2 in H1975 cells or treatment of MDA-MB-231 cells with TF-targeted antibody both inhibited lung metastasis (10, 25). The TF-induced fibrin deposits in the A549 tumors facilitated immune-suppressive tumor microenvironment via the recruitment of myeloid-derived suppressor cells (21).

**FIGURE 7:**
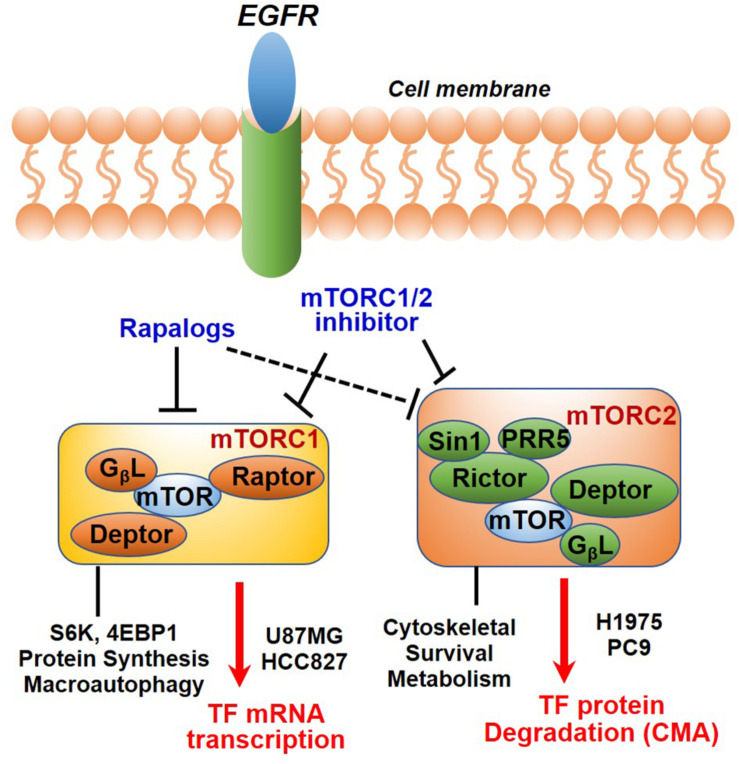
Diagram showing EGFR to mTOR signaling network and effects of mTOR inhibitors on TF regulation. In EGFR-mut cancer cells, mTOR kinase inhibitor acutely targets both mTORC1 and mTORC2, while rapalog primarily targets mTORC1 but can also disrupt mTORC2 upon a prolonged treatment. As shown, mTOR inhibitors differentially modulate TF expression in a cellular context-dependent manner. mTORC1 promotes TF mRNA transcription in HCC827 and U87MG cells; An mTORC2-dependent suppression of chaperon mediated autophagy (CMA) mechanism is proposed for up-regulation of TF protein in H1975 and PC9 cells.

In summary, we have identified TF as a new molecular and functional target of mTOR. Our results together with previous studies highlighted a new role of mTOR complexes in EGFR-mut NSCLC progression and therapy resistance. Downregulation of mTOR-TF axis activity likely mediates the therapeutic mechanism of mTORC1/2- and TF-targeted agents (Figure 7).

## Data Availability Statement

The raw data supporting the conclusions of this article will be made available by the authors, without undue reservation.

## Ethics Statement

The animal study was reviewed and approved by the Institutional Animal Care and Use Committee of Fudan University.

## Author Contributions

YCo and KY: conception and design, writing, review, and/or revision of the manuscript, administration, technical, material support, and study supervision. YCo, QL, and YCh: development of methodology. YCo, QL, XZ, and YCh: acquisition of data, analysis, and interpretation of data. All authors contributed to the article and approved the submitted version.

## Conflict of Interest

The authors declare that the research was conducted in the absence of any commercial or financial relationships that could be construed as a potential conflict of interest.

## Supplementary Material

FIGURE S1 Effect of mTOR inhibitor treatment on tumor vasculature. **(A,B)** Tumor sections of H1975 **(A)** or U87MG **(B)** were analyzed by IHC staining with anti-CD31 or co-IF staining with anti-CD31 (green) and anti-α-SMA (red). Representative images are shown (left panels) and quantified (right panels), ***P* < 0.01.

FIGURE S2 Effect of TF-targeted antibody SC1 on tumor growth. Cells of U87MG (5 × 10^6^) were mixed with 100 μg mouse IgG or 100 μg SC1. The cell/antibody mixture was subcutaneously (s.c.) implanted into flank area of female nude mice (*n* = 8). Tumor growth curves are shown.
