# Supplementary figures and images for: mTOR Promotes Tissue Factor Expression and Activity in EGFR-Mutant Cancer
Source: Front Oncol. 2020 Aug 14;10:1615. doi: 10.3389/fonc.2020.01615 (PMC7456926; doi:10.3389/fonc.2020.01615)

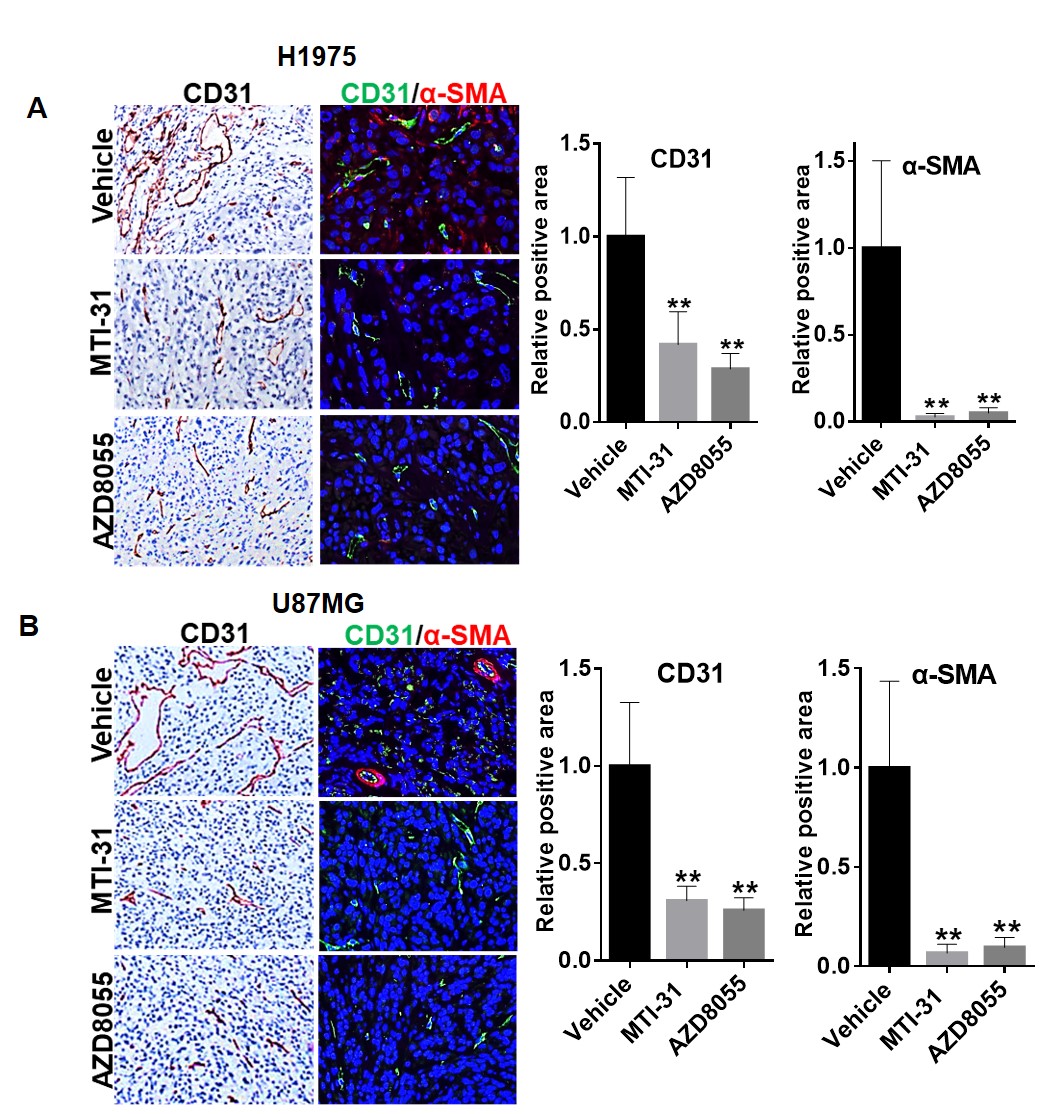

Supplement: FIGURE S1 — Effect of mTOR inhibitor treatment on tumor vasculature. (A,B) Tumor sections of H1975 (A) or U87MG (B) were analyzed by IHC staining with anti-CD31 or co-IF staining with anti-CD31 (green) and anti-α-SMA (red). Representative images are shown (left panels) and quantified (right panels), **P < 0.01. [file Image_1.JPEG]

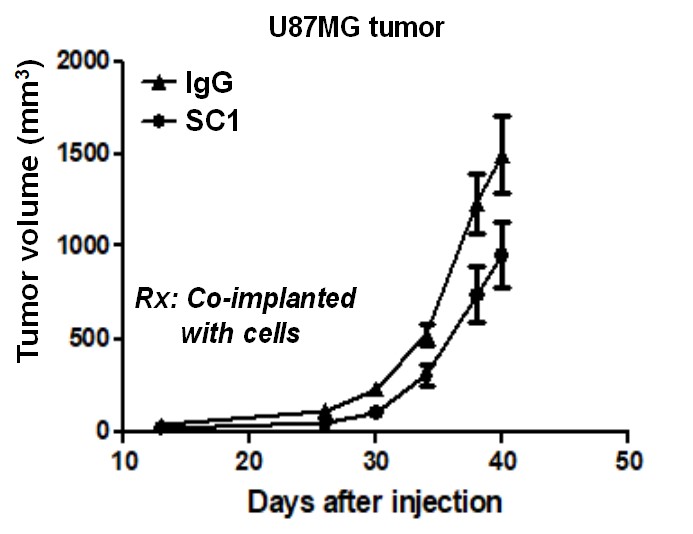

Supplement: FIGURE S2 — Effect of TF-targeted antibody SC1 on tumor growth. Cells of U87MG (5 × 10^6^) were mixed with 100 μg mouse IgG or 100 μg SC1. The cell/antibody mixture was subcutaneously (s.c.) implanted into flank area of female nude mice (n = 8). Tumor growth curves are shown. [file Image_2.JPEG]
